# Analysis of the Appropriateness of the Use of Peltier Cells as Energy Sources

**DOI:** 10.3390/s16060760

**Published:** 2016-05-25

**Authors:** Radovan Hájovský, Martin Pieš, Lukáš Richtár

**Affiliations:** Department of Cybernetics and Biomedical Engineering, VSB-Technical University of Ostrava, Ostrava 70833, Czech Republic; radovan.hajovsky@vsb.cz (R.H.); lukas.richtar.st1@vsb.cz (L.R.)

**Keywords:** energy source, experiments, monitoring, Peltier cells, temperature measurement

## Abstract

The article describes the possibilities of using Peltier cells as an energy source to power the telemetry units, which are used in large-scale monitoring systems as central units, ensuring the collection of data from sensors, processing, and sending to the database server. The article describes the various experiments that were carried out, their progress and results. Based on experiments evaluated, the paper also discusses the possibilities of using various types depending on the temperature difference of the cold and hot sides.

## 1. Introduction

Peltier cells are based on the principle of thermoelectric phenomena. These phenomena are based on the formation of a difference of voltage levels at the junctions of two different metal materials. The first important discovery relating to thermoelectricity occurred in 1821 when a German scientist, Thomas Johann Seebeck, found that an electric current would flow continuously in a closed circuit made up of two dissimilar metals provided that the junctions of the metals were maintained at two different temperatures [1]. Thirteen years later, the French physicist Jean Charles Peltier discovered an effect of release or absorption of the heat in the interconnection of two different wires under a current flow. Whether the heat is released or absorbed and in what amount depends on the current which passes through the circuit. The Seebeck and Peltier effects are mutually inversed. Later on, a German physicist Edmund Altenkirch started to investigate this problem after some time. He showed an interest in thermoelectric generators (TEG) beginning in 1909, and already began to calculate their efficiency quite precisely within the same year. A year later, in 1910, he also examined the thermoelectric coolers (TEC), finding that previously used materials had been unsuitable at all. He discovered that semiconductors are much more appropriate materials, both for thermoelectric coolers and thermoelectric generators [2].

Commercial use was introduced in the 1950s, and it was mainly used in military and space technology for electrical power generators, cooling and for precise temperature control as well. Only in recent years have the cells using this phenomenon (Peltier cells) begun to be used in civilian sectors [3,4].

**Figure 1:**
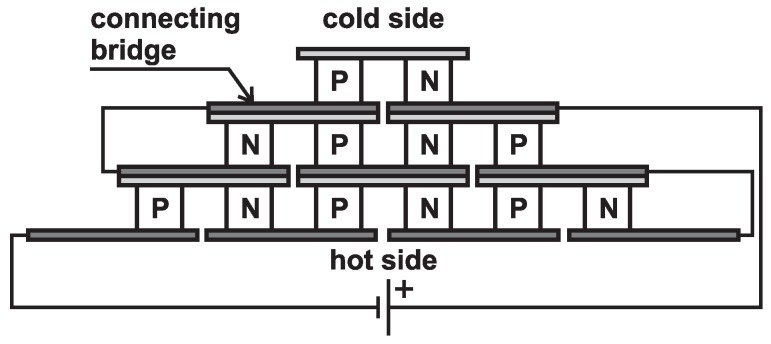
Serial interconnections of the cells.

Peltier cells are composed of three parts: a P-type semiconductor, N-type semiconductor and a connecting bridge (see Figure 1). Particular semiconductors are bound by a connecting bridge that also brings the electric energy for them (while it also absorbs the heat energy from the Peltier cells). Individual cells can be attached in series. Peltier cells that are used for various applications in practice are composed of several elementary cells. Peltier cells can be divided into two main areas, particularly for cooling and for generation of electric energy [5,6,7,8].

### 1.1. Cells for Cooling

Cells can work if we supply the necessary current that will pass through them. Then, one of the joints of the thermocouple will start heating and the second one will start cooling. This is used in small devices that cannot use compressor-type cooling, for example, because of the size or position, *etc*. Another advantage may be practically zero noise that cannot be achieved in compressor cooling devices. One example of using such an application may be cooling in portable refrigerators used in cars.

### 1.2. Cells for Generation of Electric Energy

Thermoelectric generators are devices that generate electric voltage due to the temperature difference. Devices generating electrical energy may either consist of a single Peltier cell or a group of them. A source of thermal energy is usually the heat generated by combustion whether fossil fuels or waste. Another source may be the heat from waste water from refrigeration devices, *etc*. In our case, we will deal with the heat radiated by the Ostrava slag dump heaps.

## 2. Problem Description

In the territory of the Moravian-Silesian region, there are large numbers of old mine dumps that are the remains of long-term mining activities. During the active underground mining activities, waste rock had been piled up over time, including coal residues. It is estimated that, in some cases, the content of coal in the tailings is up to 40%. Given this large volume of coal substances, thermal processes started to develop in some basins. They are characterized by progressive burning inside the mine dump along with negative side effects from these processes. Mining dumps form large territories, some of which currently host existing buildings or serve as tourist destinations. The above-mentioned thermal processes have hugely negative impacts which have adverse effects on the surrounding environment. The most important include the burning of vegetation, dust, presence of dangerous gases, formation of burnt areas, *etc*. For these reasons, it is necessary to implement a long-term monitoring of the symptoms of thermal processes. The basic measured quantities are temperature and concentration of hazardous gases (CO, CH${}_{4}$) [9]. Measurements can be performed either manually or via automated measurement systems. Use of measurement systems consisting of custom sensors of given quantities and a superior system (mostly telemetry stations) raises the requirement for autonomous power supply due to the absence of 230V power supply. Thus, all systems are powered by batteries. This solution is not ideal because the batteries will eventually become discharged due to current load. The classic solution involving using solar panels for charging is impossible because of the immediate destruction or theft caused by antisocial persons. As the monitoring mainly covers extensive thermal processes where temperatures can reach up to several hundred ${}^{\circ }C$, an idea was proposed of using the features of Peltier cells and their energy to recharge the batteries [9,10,11]. A usability study of individual types of Peltier cells depending on the temperature difference is discussed in this paper.

## 3. Ideological Conceptual Design

**Figure 2:**
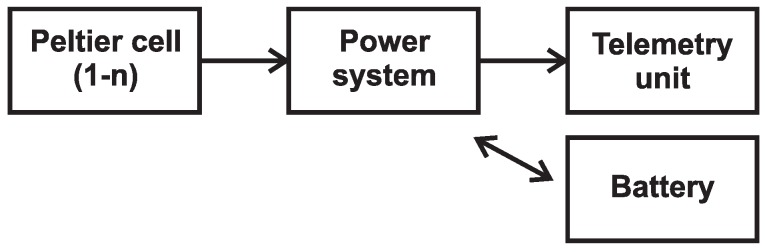
Block scheme.

**Figure 3:**
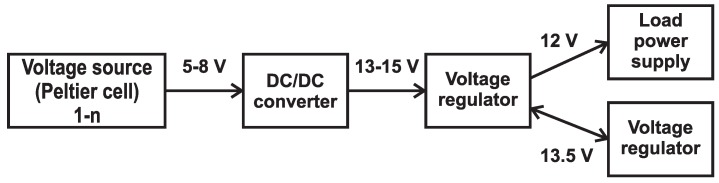
Block scheme of power supply system.

As was mentioned above, for a long-term autonomous operation of the measurement system, it is necessary to design a system to charge the battery pack. The proposed system uses the properties of Peltier cells and provides either permanent power or continuous battery charging in the case of small temperature differences [11,12]. A general diagram of the proposed system is shown in Figure 2. A block diagram of the proposed system is then shown in Figure 3.

The power supply system consists of five parts. The first part is a voltage source, which represents a system of several cells with at least 5V output voltage. This voltage is already sufficient for the DC/DC converter that boosts the DC input voltage to DC 13–15V. For better and more efficient operation of the power system, it is necessary to use a voltage regulator, which provides either battery charging or load power supply if the battery is charged. Simultaneously, it keeps monitoring and indicating the status of the battery to prevent overcharging and total discharge [13].

## 4. Experimental Verification of Efficiency of Peltier Cells

The first part of the efficiency analysis is to determine the output voltage and current within TEG and TEC depending on the temperature difference between the hot and cold sides and comparison of three selected kinds of cells. Another part of the analysis is verification of the operation of the power system under ideal conditions and then under simulated real-world conditions.

The first type was a TEG Peltier cell. This one was purchased from China, and there is not any information which would determine the type of cell, the power, maximum voltage, temperature *etc*. This TEG Peltier cell will be hereinafter denoted as TEG. The second type was the TEC Peltier cell TEC MCTE1-12715L-S, hereinafter referred to as TEC1. The third type was TEC Peltier cell TEC-12710, hereinafter referred to as TEC2. TEC1 has maximum power of 145W at $\Delta T=75{\;}^{\circ }C$, and TEC2 has maximum power of 96W at $\Delta T=75{\;}^{\circ }C$. Materials in the semiconductors that are used in these Peltier cells are not specified by the manufacturer.

During the first four experiments, K-thermocouples and Pt100 resistance temperature sensors were used for temperature measurement. Accuracy of the measurements of these temperatures was determined by the type of sensor. The tolerance of the K-thermocouples is $2.2{\;}^{\circ }C$, and for Pt100, it is $0.1{\;}^{\circ }C$.

### 4.1. The First Experiment

**Figure 4:**
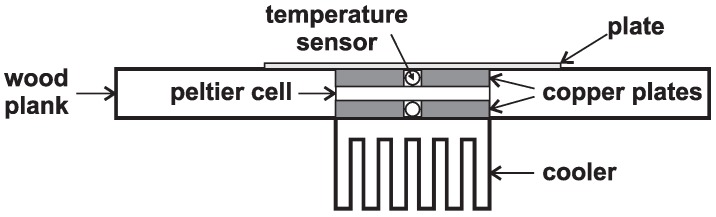
Layout of the first experimental measurement.

The first experiment involves the measurement when the hot side is heated with a heat gun, and the cold side is cooled by a passive heat sink of the central processor unit (CPU). Separation of both sides is partly ensured by a thin wooden board with a hole in the shape of the Peltier cell. This hole is then fitted by the cell and both sides are attached with copper plates that are located between the cell and cooler (*i.e.*, a heated plate). A sensor for temperature measurement is inserted between copper plates on each side of the board, while the cooler is bolted from one side and the heated plate is on the other side (see Figure 4).

The initial temperature of the first experimental measurements with TEG cells was $18{\;}^{\circ }C$, which was measured using two temperature sensors (Pt100 and K-thermocouple attached to the multimeter UNI-T UT33C). In this experimental attempt, maximum voltage of 0.55V was achieved at a temperature difference of $65{\;}^{\circ }C$. The dependence of voltage on Peltier cells on the temperature difference between heated and cold sides increases almost logarithmically up to the values of 30–$40{\;}^{\circ }C$, where the increase becomes milder than the voltage during the remaining part of the measurement. Table 1 summarizes results of the first experiment.

The problem identified by experimental measurements is the cooling developed by the cells which is a width of only 4mm. This leads to a vast heat transfer through the entire cell. Another problem of this experiment was poor heat transfer through copper plates. Further experimental measurement is therefore provided with water cooling.

### 4.2. The Second Experiment

**Figure 5:**
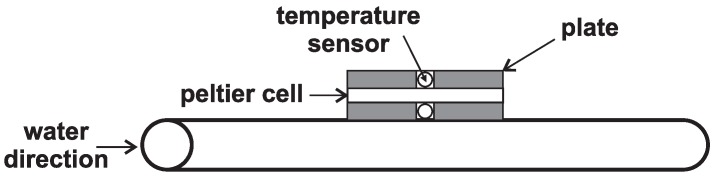
Layout of the second experimental measurement.

Cooling during the second measurement is solved by use of iron square size of 50mm×50mm, which is welded to an iron pipe that is flattened in the middle to make a large contact area with the iron square. This designed cooler drives the water from the tank by a pump towards the other side where it is let out of the tank again. Part of the cooler is a canal in the iron square that holds Pt100 temperature sensors needed to measure the cold side of the cell. The other side of the cell is overlapped by an aluminum plate in a square shape with a canal for the second temperature sensor (see Figure 5).

The initial temperature for experiment number two measured by both temperature sensors was $13{\;}^{\circ }C$. Since much higher temperature differences were achieved during this measurement, the output voltage was also larger. A difference of about $150{\;}^{\circ }C$ caused approximately 2.5V per cell. Measured values gained during the second experimental measurements indicate that the voltage depending on the temperature difference increases logarithmically, as in the first case. During no-load current measurement, the cell voltage dropped to about 1.5V, and 800mA was measured by an ammeter. Table 2 summarizes the results of the second experiment.

### 4.3. The Third Experiment

Using the same way there was the third experimental measurements of TEC Peltier cell although designed to cool at which is most efficient but it also works as a thermoelectric power generator with lower efficiency.

Better results were achieved paradoxically with TEC cells that are primarily designed to cool. Unlike previously measured thermoelectric generators with a size of 40mm×40mm×4mm, this time, the size of the cell is 50mm×50mm×4mm. The maximum achieved voltage was 3.5V and, during no-load current measuring, the voltage dropped to 1.8V at 1A flowing current.

Voltage of these cells depending on the temperature difference is not yet increasing logarithmically. As in the case of thermoelectric generators, it is rather exponential until 2V when the voltage begins to rise more slowly, nearly linearly. Table 3 summarizes results of the third experiment.

### 4.4. The Fourth Experiment

**Figure 6:**
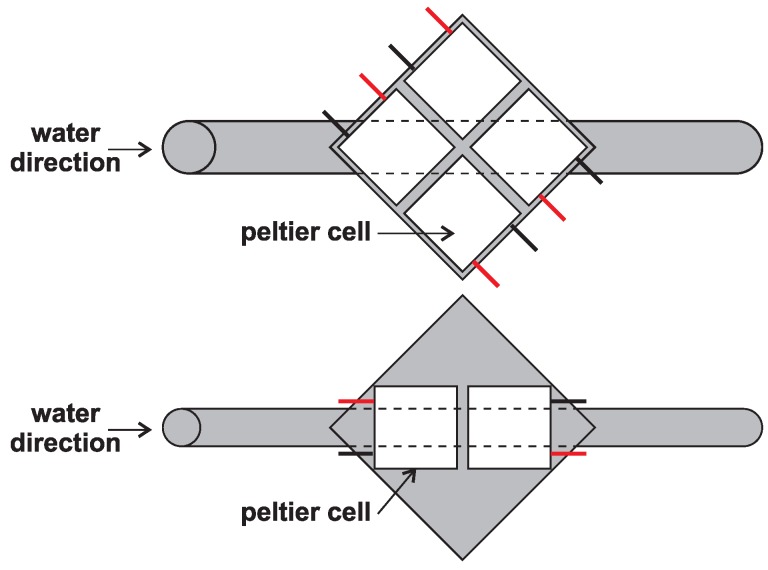
Experimental setup for placement of more cells: (**Up**) Four cells; (**Down**) Two cells.

Another test for the analysis of the Peltier cells is similar to the second and third experimental measurements with the difference that the surfaces for heat transfer are adapted to mount the four cells at one time (see Figure 6). The reason for this experiment is the possibility to connect more cells in various ways, either in series, parallel, or different combinations.

When assembling four TEG cells with $25{\;}^{\circ }C$ on the cold side and $55{\;}^{\circ }C$ on the hot side, the voltage per cell reached about 700mV, thus the value possible to be achieved when connecting cells in series is 4V minimum. This voltage is sufficient, since there are converters that can lift this DC voltage up to 15V DC, which can be either stabilized at the level appropriate for charging a 12V battery or regulated for battery charging or for direct power supply for the load.

Upon heating (or cooling), the temperature difference increased and so did the voltage in cells. However, at a temperature difference of $40{\;}^{\circ }C$, the DC/DC converter increased the voltage to 16V, which thus entered into the voltage regulator. After measuring the voltage by a voltmeter at terminals determined for the battery, the measured value was 12.05V. To charge a 12V battery, however, it should be at least 13.5V voltage. This undesirable voltage drop is caused by low output current of the cells. Power of the cells is almost consumed on operating the inverter and controller, as their operation is possible thanks to the power of the cell without using an external power source.

Another problem came up with this connection, that is, if the temperature of the hot side has risen above the value of $100{\;}^{\circ }C$, voltage of 3.5V almost immediately dropped to 0V and held at such low levels up until the temperature dropped below $70{\;}^{\circ }C$. Subsequently, voltage again rose to approximately 1.5V (temperature difference diminished). If the cells were turned upside down, they again generated electricity again, but the polarity was reversed (red wire—negative pole, black wire—positive pole). Upon heating (or cooling) voltage kept rising again, but if the temperature of the cold side rose above $80{\;}^{\circ }C$ (temperature of the hot side was $120{\;}^{\circ }C$ and, due to the small width of the article, there was a large heat transfer through the cell), the voltage dropped again to almost 0V and rose again after the cold side temperature dropped below $60{\;}^{\circ }C$. This can be explained by the fact that the cells are designed for a given temperature range, and exceeding this range leads to the voltage drop. The reason why the initial experiments showed such high measured voltages is a bad heat transfer between the heated part and cell plate.

The problem with a small current output of the cells may be fixed by parallel connection of the cells of TEG or TEC2 types. Regarding such parallel interconnection, it is important to have the voltage the same in both cells, which is difficult to achieve since each cell is located in another part of the jig. If the voltage is different, the current begins to flow between the cells (one begins to behave as a source and the other as a load). In this situation, it is possible to achieve higher current than when using a single cell, but this current will be less than the sum of both currents of the cells.

When using a series interconnection, the output current depends on the smallest current flow of individual cells. When installing four articles on the above-mentioned jig, there is no significant difference at particular values of the current.

An interesting idea is the installation of several cells on top of each other due to heat transfer. Cells located on the cold side have their other sides heated due to the heat transfer through cells located on the hot side; thus, cells on the cold side have such a temperature for the voltage drop to almost 0V.

Such placed cells (cold side—TEG, hot side—TEC) have been interconnected in several ways. Firstly, pairs of adjacent cells have always been connected in parallel and the resulting four pairs connected in series. Secondly, there were parallel connections of two of the same type of cells (TEC and TEG) and subsequent serial connection of such formed pairs. Another interconnection involved four cells of the same type in parallel and subsequent serial connection of quaternions.

However, none of these connections reached the needed values required due to above-mentioned reasons, since the current and voltage levels were never the same.

This experiment using four cells revealed the biggest problem due to the uneven cooling of all cells, and therefore it is not appropriate to order them in a series (overall current is then equal to the current of the worst cell) or in parallel (different voltages cause current flowing between the cells, thus reducing power).

In the case of placing two TEC1 cells into the jig, the result was much better, since the temperature of both sides of the cell is very similar. The jig used in the previous experiment is made so that a tube is welded to the diagonal of the iron square, thus the greatest cooling occurs in these locations. Cells were placed on this diagonal to achieve the desired temperature difference.

Desired output voltage of two cells connected in a series was achieved at a temperature difference of $50{\;}^{\circ }C$. This temperature difference is achievable at mining dumps since the temperature inside the heaps reach even up to $1500{\;}^{\circ }C$, and, below the surface, it can still be about $80{\;}^{\circ }C$.

Short circuit current of the cells was 1.6A, and, after connecting the DC/DC converter, there was short circuit current of about 1A at the terminals of the battery regulator. The voltage at the terminals for the battery connection was enough to charge the battery even in the case of connection of the load in the form of a 12V computer fan.

This experiment indicates that if the temperature difference reaches $50{\;}^{\circ }C$, it is possible to reach the required power with the use of TEC1; thus, it is possible to use this power supply system.

## 5. Simulation of Real Operation at the Mining Dump

Simulation of the conditions within the mining dump is carried out using a plastic barrel filled with fine gravel, which is heated by power resistors and into which an aluminum profile is inserted.

**Figure 7:**
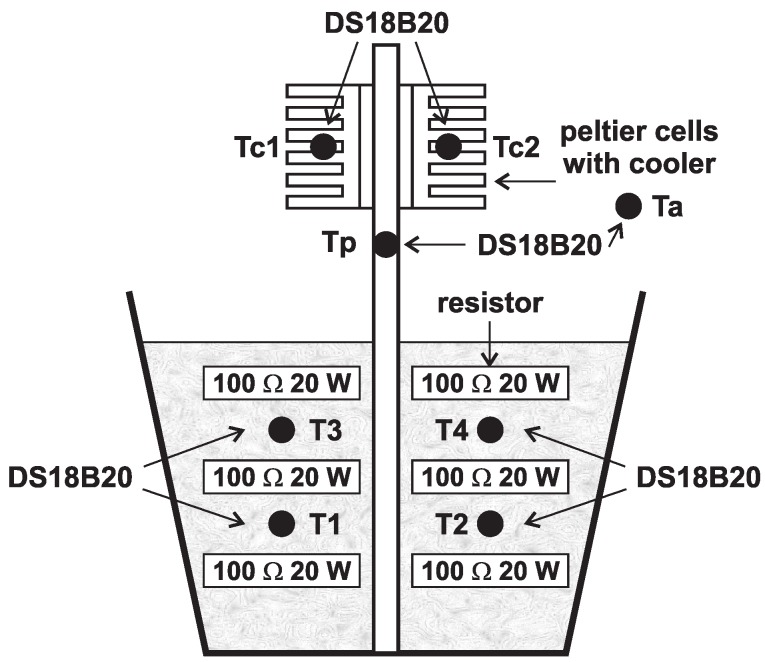
Layout of experimental setup for simulation of real conditions.

This profile holds four Peltier cells with passive coolers or passive cooler fitted with fans (see Figure 7).

**Figure 8:**
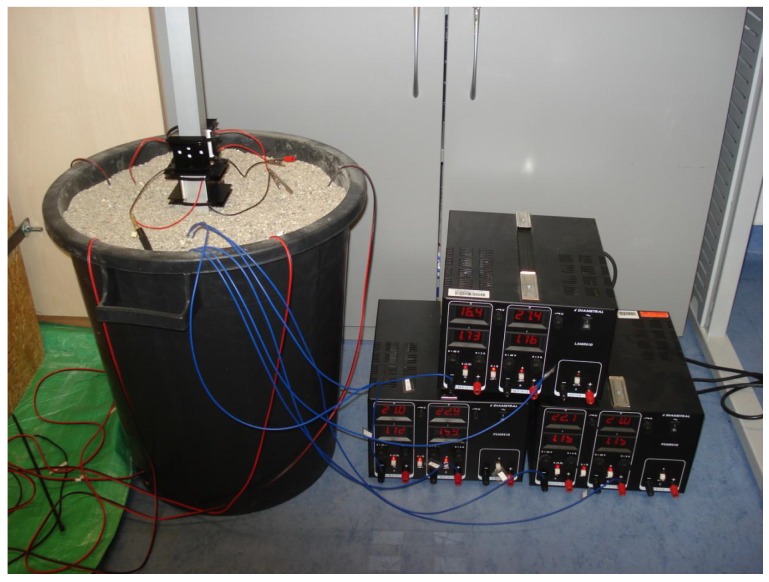
Photography of experimental setup for simulation of real conditions.

Resistors are connected in parallel due to current flow separation (as opposed to voltage division by the serial connection). Resistors are located in a barrel in three layers. Each layer contains four resistors (from all four sides of the aluminum profile). Each branch of the resistors is supplied separately by voltage supply U=44.7V and the current I=1.78A. Power of such interconnected resistors reached almost 80W, see Figure 8.

**Figure 9:**
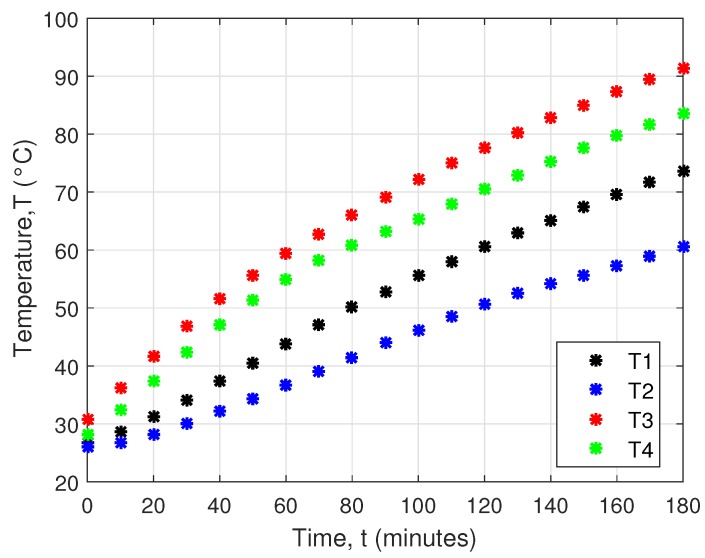
Measured soil temperatures.

Simulation of the real conditions used V7132 coolers for cooling, which are 50mm wide and 21mm high. However, measurement under simulated conditions showed that these coolers are not suitable, because they are incapable of providing enough cooling of the cells. Figure 9 shows measured temperatures in soil.

An experiment with simulation of real conditions used digital temperature converters DS18B20 having a quantization step at 12-bit resolution as small as $0.0625{\;}^{\circ }C$, but the manufacturing tolerance is $0.5{\;}^{\circ }C$.

**Figure 10:**
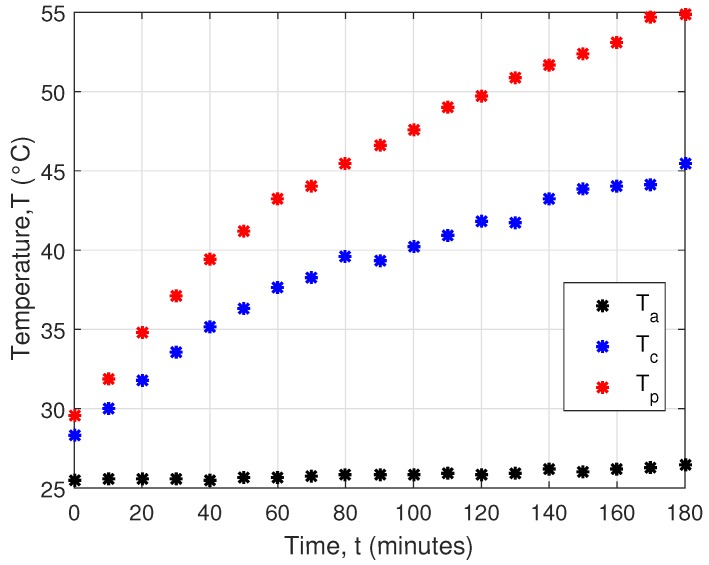
Measured temperatures on the hot and cold sides of Peltier cell.

Heat loss during heat transfer between soil and the aluminum profile shows Figure 10. $T_{a}$ is ambient temperature, and $T_{c}$ is average temperature computed from each cooler temperature sensor. $T_{p}$ is temperature of aluminum profile. Temperature of this profile was nearly $60{\;}^{\circ }C$, and, after a few hours of measurement, the cooler temperature decreased by less than $10{\;}^{\circ }C$.

**Figure 11:**
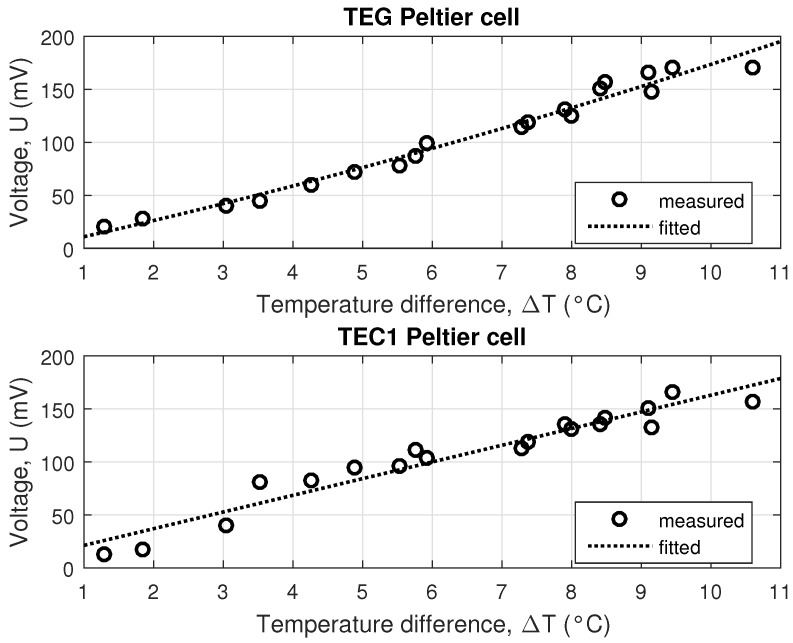
Dependence of the temperature difference on voltage in TEG and TEC1 Peltier cells for experiments simulating the real conditions at the mining dump.

The resulting voltage generated in this experiment was about 160mV in one cell, irrespective of whether it was a TEC1 or TEG Peltier cell, see Figure 11.

**Figure 12:**
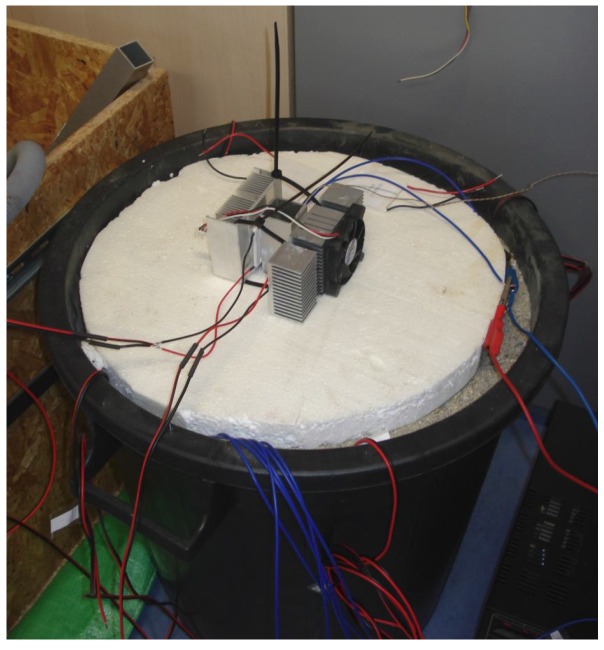
Photography of modified experimental setup.

Better results were obtained if the gap between the cooler and the gravel was filled by polystyrene since the cooler was not only heated by heat transfer through the cell but also by the ground (see Figure 12).

A proposal to solve this problem is the use of larger heat sinks, or alternatively the use of a cooler with a fan. Further measurements therefore used the CPU coolers. One cell used a cooler with a size of 110mm×50mm×30mm, which was attached to the fan with a size of 50mm×50mm. After installing the cooler, cell voltage immediately rose to 300mV and steadily kept this size. A Peltier cell mounted on an opposite wall profile was attached with to a heat sink of size 80mm×80mm×30mm, suffering from the same problem as in the case of a smaller heat sink V7132. After turning on the fan of size 80mm×80mm, the cell voltage began to rise up to 250mV, but then a problem occurred in which the fan began to cool the aluminum profile; therefore, the temperature difference again started decreasing and voltage in the first cell dropped. After turning off the bigger fan, the difference in the first cell began to increase and so did the voltage.

**Figure 13:**
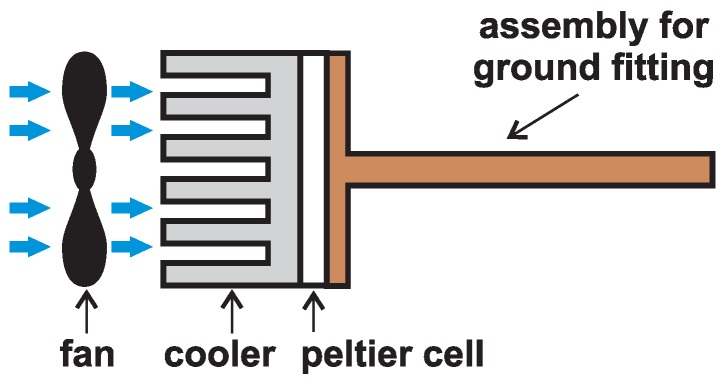
Assembly for ground fitting.

Although the result was better, it was not a significant difference. Voltage increased by 100mV maximum. Better results for obtaining the temperature difference between the aluminum profile and condenser were achieved in another way; thus, it was necessary to construct a special jig. An iron pipe with a diameter of 20mm and approximately 300mm long was welded to an iron plate in the shape of a rectangle with holes in the corners (for mounting the cooler). A Peltier cooler including a cooler was then attached to the iron plate. A thin layer of paste for good heat transfer was inserted between the plate and the cell, and also between the cell and the cooler. Subsequently, the tube was planted in the ground up to the cooler so that the plate would be deep in the ground as much as possible to avoid cooling caused by the hot side by the surrounding air. Cooling is ensured by the cooler with a fan again (see Figure 13).

The above-mentioned way resulted in a voltage gain of approximately 0.5V per cell, which is almost five times more than the previous way when simulating real-world conditions.

An interesting idea from the measurement is also rotation of the coolers determining whether to circulate cool air towards the cooler or whether to suck the warm air from the cooler and blow it out. Expected results from that idea supposed that the second way would get better results, but the implemented experiment verified that the cooler cools down better when the fan blows ambient air into the cooler, and it subsequently passes through the cooling fins, leaving through the side parts of the cooler.

Voltage obtained with the device fitted in the ground exceeded the limit of 0.5V by a few millivolts. This way could lead to achievement of resulting voltage of 3.5V for a series connection of cells, but the disadvantage is that cells located closer to the power resistors reached significantly higher voltage values than the ones located farther from the resistors.

## 6. Summary of the Experiments

Best results were obtained with TEC1 cells. Although these cells are designed mainly for cooling, they can also work as generators but with less efficiency than in the case of cooling. The other two kinds of cells did not achieve such results, and, although there were two different kinds of cells (TEC2 and TEG), the results were very similar. Price of the cells thus corresponded to their quality, which was more than twice as high in the case of TEC1 compared to the other two types. Cell size may also play a role. TEC1 cells have dimensions of 50mm×50mm×4mm unlike the other two cells with dimensions of 40mm×40mm×4mm.

A thermoelectric generator used as an electric power source to supply the measurement units is suitable in the case of a good difference of heat and under the conditions that the temperature difference is transferred to the surfaces of the cells. This finding is very important because the heat transfer through the cell is large due to the small width of the cell. The maximum measured voltage on TEC1 cells reached up to 3.5V at a temperature difference of $129{\;}^{\circ }C$. Under the load, the voltage dropped in half, but the current through the cell was more than 1A. Connection of these two cells in a series provides sufficient voltage that can be increased by the DC/DC converter enough to be capable of charging a 12V battery. The output of the convertor is moreover connected to the voltage regulator, which is responsible for monitoring the state of the battery and controls whether to charge the battery or to power the load. In the case of overcharging, it is necessary to stop charging and, in the case of discharging, to prevent it from further battery consumption to avoid irreparable damage.

In the case of simulation of real-world conditions, the results achieved were not quite close to ideal results gained under laboratory conditions, but in the case of use of a larger heat sink, it is possible to achieve greater differences in temperature and thus a greater voltage. Greater difference can also be achieved at lower ambient temperature (e.g., in the winter at $-20{\;}^{\circ }C$) or at better weather conditions.

## 7. Conclusions

The first four experiments were designed to test the ability of the Peltier cells (TEG, TEC1 and TEC2) to generate DC voltage and current. During these experiments, these thermocouples were exposed to their physical limits, being in danger of destruction. These experiments determined amplitudes of voltage and current achievable theoretically when deployed under real operation.

Simulation of real conditions showed a possibility to achieve small voltage amplitudes insufficient for recharging the battery. Some improvement occurred in the case of using a different shape of heat sink and isolation of heated soil from the cooler. The measurement results lead to the conclusion that a 12V battery can be recharged by Peltier cells but at the cost of overloading these cells. It is possible to connect more Peltier cells in a series, but this proportionally increases the cost of this alternative energy source. The overall demands on the number of Peltier cells could be reduced by adding additional sources of energy, such as solar cells.

A good alternative to the use of this approach may lie in the possibility to power wireless sensors with minimal energy demands. Such sensors usually have an average consumption of tens of μA. Using special circuits with the possibility to increase the voltage sufficiently to around 1.8–3.3V would allow the use of thermal potential at the mining dump to power these sensors.

Long-term monitoring of temperatures occurring at thermally active mining dumps indicates that the thermal potential would be sufficient to power wireless sensors that we have currently deployed at these mining dumps.

In the near future, it is also planned to verify the power system in practice in the mining dumps. This measurement must be performed repeatedly under different climatic conditions in order to investigate the feasibility of the power system used year-round and continuously. Related to this issue is a question of how much and how fast the temperature will change in the location of measurement apparatus.

Future work will also involve other alternative energy sources, and energy harvesting in general, where, in addition to the thermoelectric effect, the basic principles are yet the piezoelectric effect, photoelectric, inductive and capacitive.

## Figures and Tables

**Table 1:** Dependence of the voltage on temperature difference in Peltier cells for the first experimental measurement.

Cold Side Temperature	Hot Side Temperature	Temperature Difference	Voltage
$\left({}^{\circ }C\right)$	$\left({}^{\circ }C\right)$	$\left({}^{\circ }C\right)$	$\left(V\right)$
18	18	0	0
23	38	15	0.05
26	51	25	0.1
30	59	29	0.14
33	67	34	0.2
37	74	37	0.22
43	84	41	0.24
43	87	44	0.25
44	105	56	0.4
53	118	65	0.55

**Table 2:** Dependence of the voltage on temperature difference in Peltier cells for the second experimental measurement.

Cold Side Temperature	Hot Side Temperature	Temperature Difference	Voltage
$\left({}^{\circ }C\right)$	$\left({}^{\circ }C\right)$	$\left({}^{\circ }C\right)$	$\left(V\right)$
13	13	0	0
38	105	67	0.7
40	184	115	1.55
44	188	144	2.2
45	198	153	2.53

**Table 3:** Dependence of the voltage on temperature difference in Peltier cells for the third experimental measurement.

Cold Side Temperature	Hot Side Temperature	Temperature Difference	Voltage
$\left({}^{\circ }C\right)$	$\left({}^{\circ }C\right)$	$\left({}^{\circ }C\right)$	$\left(V\right)$
13	13	0	0
15	48	33	0.86
18	85	67	1.3
26	134	108	1.8
38	152	114	2.02
40	169	129	3.5

## Abbreviations

The following abbreviations are used in this manuscript:

CH${}_{4}$	Methane
CO	Carbon monoxide
CPU	Central processing unit
DC	Direct current
DS18B20	1-Wire digital thermometer
Pt100	Platinum resistance thermometer
TEC1	Thermoelectric cooler—type MCTE1-12715L-S
TEC2	Thermoelectric cooler—type TEC-12710
TEG	Thermoelectric power generator—unknown type

## References

[1] Seebeck T.J. (1826). Ueber die magnetische Polarisation der Metalle und Erze durch Temperatur-Differenz. Ann. Phys..

[2] Min G., Rowe D.M. (2006). Experimental Evaluation of Prototype Thermoelectric Domestic-Refrigerators. Appl. Energy.

[3] LaGrandeur J., Crane D., Hung S., Mazar B., Eder A. Automotive Waste Heat Conversion to Electric Power using Skutterudite, TAGS, PbTe and BiTe. Proceedings of the 25th International Conference on Thermoelectrics (ICT’06).

[4] Hajovsky R., Vojcinak P., Pies M., Koziorek J. Thermal Response Test (TRT)—System for Measurement of Thermal Response of Rock Massif. Proceedings of the 12th IFAC Conference on Programmable Devices and Embedded Systems.

[5] Goldsmid H.J. (1964). Thermoelectric Refrigeration.

[6] Goldsmid H.J. (1986). Electronic Refrigeration.

[7] Goldsmid H.J., Rowe D.M. (2007). Conversion Efficiency and Figure-of-Merit. Handbook of Thermoelectrics.

[8] Riffat S.B., Ma X. (2003). Thermoelectrics: A Review of Present and Potential Applications. Appl. Thermal Eng..

[9] Pies M., Hajovsky R., Ozana S. (2013). Autonomous Monitoring System for Measurement of Parameters of Heat Collection Technology at Thermal Active Mining Dumps. Elektron. Elektrotech..

[10] Hajovsky R., Pies M., Ozana S., Hajovsky J. Heat energy collection from thermally active mining dump Hedvika. Proceedings of the IEEE International Conference on Automation Science and Engineering.

[11] Bilek O., Krejcar O. Possibility of using embedded sensors of smart devices for augmented reality application. Proceedings of the 10th International Conference on Future Information Technology.

[12] Prauzek M., Musilek P., Watts A.G., Michalikova M. (2014). Powering environmental monitoring systems in arctic regions: A simulation study. Elektron. Elektrotech..

[13] Vanus J., Novak T., Koziorek J., Konecny J., Hrbac R. The proposal model of energy savings of lighting systems in the smart home care. Proceedings of the 12th IFAC Conference on Programmable Devices and Embedded Systems.

